# Association of Mediterranean Diet Adherence with Self-Reported Muscle-Related Functional Status and Fracture Probability in Postmenopausal Women Evaluated for Osteoporosis: A Cross-Sectional Study

**DOI:** 10.3390/nu18162629

**Published:** 2026-08-12

**Authors:** Cecilia Oliveri, Herbert Ryan Marini, Nunziata Morabito, Letteria Minutoli, Giovanni Squadrito, Silvia Migliaccio, Agostino Gaudio, Antonino Catalano

**Affiliations:** 1 Department of Clinical and Experimental Medicine, University of Messina, 98125 Messina, Italy; hrmarini@unime.it (H.R.M.); nmorabito@unime.it (N.M.); lminutoli@unime.it (L.M.); gsquadrito@unime.it (G.S.); 2 Department of Experimental Medicine, University Sapienza of Rome, 00161 Rome, Italy; silvia.migliaccio@uniroma1.it; 3 Department of Clinical and Experimental Medicine, University of Catania, 95123 Catania, Italy; agostino.gaudio@unict.it

**Keywords:** Mediterranean diet, sarcopenia, muscle function, SARC-F, fracture risk, osteoporosis, postmenopausal women

## Abstract

**Background:** Osteoporosis and sarcopenia frequently coexist in older adults and jointly contribute to increased risk of falls and fragility fractures. While adherence to the Mediterranean diet (MD) has been broadly associated with favorable health outcomes, its potential role in modulating muscle-related functional status and, indirectly, fracture risk remains incompletely understood. **Objective:** To investigate the association between adherence to MD and SARC-F-assessed functional status, and to explore their interplay with FRAX-estimated fracture probability in postmenopausal women evaluated for osteoporosis. **Methods:** Adherence to the MD was assessed using the Medi-Lite questionnaire. Muscle-related functional status was evaluated through the SARC-F questionnaire and objectively measured by handgrip strength. Fracture probability was estimated using the FRAX tool. Correlation and multivariable analyses were performed to evaluate associations among study variables. In addition, an exploratory indirect-effect analysis was conducted to investigate whether functional status might represent a potential intermediate pathway linking MD adherence and FRAX-estimated fracture probability. **Results:** A total of 96 participants (median age 66 years) were consecutively recruited. Greater adherence to the MD was associated with lower SARC-F scores (r = −0.292; *p* = 0.005), indicating better muscle-related functional status. SARC-F scores were inversely correlated with handgrip strength (r = −0.264; *p* = 0.013) and positively correlated with the estimated 10-year probability of major osteoporotic fractures (r = 0.289; *p* = 0.005) and hip fractures (r = 0.277; *p* = 0.008). No significant associations were observed between MD adherence and FRAX-estimated fracture probability, bone mineral density, or prevalent fractures. In the exploratory indirect-effect analysis, a significant indirect association between MD adherence and FRAX-estimated fracture probability through SARC-F score was observed, whereas the direct association was not significant. **Conclusions:** In postmenopausal women referred for osteoporosis evaluation, greater adherence to the MD was associated with better self-reported muscle-related functional status but not directly with FRAX-estimated fracture probability. These findings are consistent with the hypothesis that functional status may represent an intermediate link between dietary habits and estimated fracture risk. However, because dietary adherence, functional status, and fracture risk were assessed simultaneously, no causal or temporal inferences can be drawn. Consequently, the indirect-effect findings should be regarded as exploratory and hypothesis-generating, requiring confirmation in adequately powered longitudinal and mechanistic studies.

## 1. Introduction

Osteoporosis and sarcopenia frequently coexist in older adults and jointly contribute to an increased risk of falls, frailty, and fragility fractures [1,2,3]. Increasing evidence indicates that muscle function, beyond bone mineral density (BMD), represents a critical determinant of fracture risk, acting through impaired balance, reduced muscle strength, and increased susceptibility to falls [4,5,6]. These observations have reinforced the concept of the musculoskeletal unit as an integrated system, in which bone and muscle are closely interconnected both mechanically and metabolically [1,3].

Beyond mechanical interdependence, the muscle–bone unit is increasingly recognized as an endocrine and paracrine network mediated by myokines, osteokines, and inflammatory cytokines [1]. Skeletal muscle secretes factors such as irisin and myostatin that regulate bone remodeling, whereas bone-derived molecules, including osteocalcin, may influence muscle metabolism and function. This bidirectional crosstalk is particularly relevant in aging, where chronic low-grade inflammation contributes to both sarcopenia and bone loss. In this context, mounting evidence highlights the role of systemic inflammatory pathways in modulating musculoskeletal decline, influencing both bone fragility and muscle performance, and ultimately increasing fracture risk independently of BMD [1,2,3]. Notably, findings from clinical and translational studies have emphasized the contribution of inflammatory molecules and systemic immune activation to fracture risk and musculoskeletal deterioration, supporting the relevance of inflammation-related mechanisms as key mediators of skeletal fragility and functional impairment in older adults [7,8,9].

These observations indicate that interventions designed to preserve muscle strength and physical performance may lead to clinically meaningful reductions in fracture risk, via mechanisms that are at least partially independent of BMD [10,11,12].

The Mediterranean diet (MD), characterized by a high intake of plant-based foods and extra virgin olive oil, is widely recognized as a key determinant of cardiometabolic health and longevity [13,14,15]. More recently, attention has shifted toward its potential role in musculoskeletal health, with growing evidence supporting beneficial effects on both bone and muscle [16,17,18,19]. These effects are likely mediated by its anti-inflammatory and antioxidant properties, as well as by the high intake of micronutrients and bioactive compounds [20,21].

Adherence to the MD has been consistently associated not only with reduced cardiovascular and metabolic risk but also with a slower decline in physical performance and frailty. Large epidemiological studies have shown that individuals with higher adherence to the MD exhibit better gait speed, muscle strength, and overall physical performance. Furthermore, longitudinal data suggest that such dietary patterns may delay the onset of disability and functional impairment, thereby indirectly influencing fall risk and fracture susceptibility [22,23].

Mechanistically, the MD may influence musculoskeletal health through multiple pathways, including optimization of vitamin D status, adequate calcium intake, and higher protein consumption, all of which are associated with attenuation of bone loss [24,25,26,27].

Nevertheless, limited evidence is available from studies simultaneously evaluating MD adherence, muscle function, and validated fracture risk prediction tools within a unified analytic framework, particularly in high fracture risk populations.

Most available studies have examined the relationship between the MD and bone-related outcomes or, separately, muscle-related parameters, whereas integrative analyses simultaneously addressing dietary patterns, muscle function, and validated fracture risk prediction tools remain scarce [27,28,29,30]. Consequently, a significant knowledge gap persists regarding whether adherence to the MD may influence fracture risk indirectly through its effects on muscle function, rather than through direct effects on bone density alone. In this context, the present study aimed to investigate the association between adherence to the MD and self-reported muscle-related functional status and to explore their relationship with estimated fracture risk in Caucasian postmenopausal women undergoing osteoporosis assessment.

## 2. Materials and Methods

This cross-sectional observational study included postmenopausal women consecutively referred to a tertiary care outpatient clinic for bone and mineral metabolism disorders at the University Hospital of Messina.

Osteoporosis and osteopenia were defined according to World Health Organization (WHO) criteria based on T-score thresholds [31,32,33], as endorsed by current clinical guidelines. Exclusion criteria included thyroid and parathyroid disorders, active or previous malignancy, severe organ failure (heart, liver, kidney, or respiratory), and chronic use (>3 months) of medications known to affect bone metabolism (e.g., glucocorticoids, aromatase inhibitors, proton pump inhibitors, antiepileptic and selective serotonin reuptake inhibitors).

All participants provided written informed consent. The study was approved by the local Ethics Committee (Prot. 71/19, date of approval 28 November 2019) and conducted in accordance with the Declaration of Helsinki.

A priori sample size estimation was grounded on the ability to detect moderate associations (r = 0.30) between adherence to the MD and functional outcomes, assuming a two-tailed significance level (α) of 0.05 and a statistical power of 80%. Under these parameters, the minimum required sample size was determined to be 85 participants. The final study cohort comprised 96 individuals, thereby exceeding the sample size estimated as necessary for the primary analyses.

Participants underwent a comprehensive clinical and biochemical evaluation, including assessment of comorbidities, medication use, and lifestyle factors (e.g., smoking status and physical activity), which are known to influence both muscle function and bone health. Physical activity was assessed through a general clinical interview: although participants were not sedentary and maintained their usual daily activities, none reported regular engagement in structured or vigorous exercise programs (e.g., gym-based training or organized sports). As levels of physical activity were generally comparable across the cohort, with limited variability, this variable was not included as a covariate in the regression models.

Fracture risk was estimated using the Fracture Risk Assessment Tool (FRAX^®^), a validated algorithm that predicts the 10-year probability of major osteoporotic fractures (clinical spine, forearm, hip, or shoulder) and hip fractures. For the purposes of this study, “fracture risk” denotes the FRAX-derived 10-year probability of major osteoporotic or hip fracture and should not be interpreted as prospectively ascertained fracture incidence.

The FRAX model integrates multiple clinical risk factors, including age, sex, body mass index (BMI), prior fragility fractures, parental history of hip fracture, smoking status, long-term glucocorticoid use, rheumatoid arthritis, secondary causes of osteoporosis, and alcohol consumption. FRAX-estimated probabilities were calculated without inclusion of femoral neck BMD, consistent with our focus on clinical, non-densitometric determinants of fracture risk; BMD and T-score were instead examined directly as separate outcomes in relation to Medi-Lite score and SARC-F. Country-specific calibration was applied using the Italian FRAX model to ensure appropriate adjustment for national epidemiological data. The resulting scores were expressed as 10-year fracture probabilities (%) and were used as continuous variables in subsequent analyses [34,35].

BMD was assessed at the lumbar spine (L1–L4) and femoral neck by dual-energy X-ray absorptiometry (DXA), using a Discovery Wi densitometer, manufactured by Hologic, Inc., headquartered in Marlborough, MA, USA. All measurements were performed by trained technicians according to manufacturer guidelines and international recommendations. Daily quality control procedures were conducted using a calibration phantom supplied by the manufacturer to ensure measurement stability and reproducibility. Participants were positioned according to standard protocols, with lumbar spine scans obtained in the supine position and femoral neck measurements performed with internal rotation of the hip to optimize anatomical alignment. BMD values were expressed in grams per square centimeter (g/cm^2^), and T-scores were calculated using the young adult reference database.

Osteoporosis and osteopenia were defined according to standard diagnostic thresholds, with a T-score ≤ −2.5 SD indicating osteoporosis and a T-score between −1.0 and −2.5 SD indicating osteopenia. For the purposes of analysis, both absolute BMD values and T-scores at each skeletal site were considered [36].

To minimize measurement variability, all scans were analyzed by the same experienced operator. Vertebrae affected by artifacts (e.g., degenerative changes, vertebral fractures, or calcifications) were excluded according to standard densitometric criteria. The coefficient of variation (CV) for repeated measurements at the lumbar spine and femoral neck was <1%.

Vertebral fractures were assessed by lateral spine radiographs and classified using the Genant semiquantitative method [37].

Self-reported muscle-related functional status was evaluated using the SARC-F questionnaire, a widely validated screening tool for identifying individuals at risk of sarcopenia [38,39]. The SARC-F includes five self-reported domains: strength, assistance in walking, rising from a chair, climbing stairs, and history of falls. Each item is scored on a 3-point Likert scale (0–2), with a total score ranging from 0 to 10 and a threshold ≥ 4 indicating a high likelihood of sarcopenia.

In this study, SARC-F was used as a measure of overall muscle-related functional status, reflecting patient-reported limitations in daily physical performance rather than direct quantification of muscle mass.

Handgrip strength was assessed using a calibrated Jamar Hydraulic Hand Dynamometer (Performance Health, Warrenville, IL, USA), according to standardized procedures. Measurements were performed with participants in a seated position, with the shoulder adducted and neutrally rotated, the elbow flexed at approximately 90°, and the forearm in a neutral position. Participants were instructed to exert maximal isometric force for a few seconds during each trial. Three consecutive attempts were performed for each participant. The highest value (kg) was retained for statistical analysis, as recommended for maximal grip strength assessment [40].

Adherence to the Mediterranean diet (MD) was assessed using the validated Italian Medi-Lite questionnaire, a literature-based scoring system designed to evaluate adherence to the main components of the Mediterranean dietary pattern. The Medi-Lite score was used to estimate participants’ usual dietary habits and overall adherence to the MD, rather than food intake over a predefined recall period. The questionnaire was administered by a trained physician through a structured interview. The questionnaire includes nine food categories: fruit, vegetables, cereals, legumes, fish, meat and meat products, dairy products, alcohol, and olive oil. Each component is scored from 0 to 2 according to consumption frequency. Protective food groups are scored positively, while foods less characteristic of the MD are inversely scored; moderate alcohol consumption is also positively scored. The total Medi-Lite score ranges from 0 to 18, with higher values indicating greater adherence [41,42,43].

Statistical analyses were performed using MedCalc software (version 10.2.0.0, Mariakerke, Belgium) and R Studio (R version 4.6.1). Data distribution was assessed using the Shapiro–Wilk test. Continuous variables were reported as median and interquartile range (IQR). Associations between variables were evaluated using Spearman’s rank correlation coefficient. Additionally, multivariable associations were assessed using multiple linear regression models, with SARC-F score and FRAX-estimated probabilities entered as continuous dependent variables and age and BMI as independent variables, to assess the robustness of the observed associations and to minimize residual confounding; standardized β coefficients, 95% confidence intervals, and *p*-values are reported. Model assumptions (linearity, homoscedasticity, multicollinearity) were checked prior to interpretation. Because age and BMI are direct components of the FRAX algorithm, regression models with FRAX score as the dependent variable and age/BMI as predictors are expected to yield high R^2^ values by construction. To isolate the contribution of SARC-F, we report the incremental R^2^ (ΔR^2^) associated with adding SARC-F to a baseline model containing age and BMI alone. In a sensitivity analysis, the association between Medi-Lite score and SARC-F was additionally adjusted for smoking status, previous fragility fracture, and family history of hip fracture, in addition to age and BMI. Because the alcohol-related item embedded in the FRAX algorithm reflects heavy alcohol intake (≥3 units/day)—a threshold not met by any participant in this cohort—whereas the Medi-Lite alcohol component reflects moderate wine consumption, a further sensitivity analysis was performed using a modified Medi-Lite score excluding the alcohol component. To address potential overlap between the alcohol component of the Medi-Lite score and the alcohol variable embedded in FRAX, a correlation analysis using a modified Medi-Lite score excluding the alcohol item was also performed, confirming the association with SARC-F was materially unchanged (r = −0.309, *p* = 0.0045). An exploratory statistical decomposition (indirect-effect analysis) with nonparametric bootstrap resampling (5000 simulations) was conducted to investigate whether SARC-F mediated the association between adherence to the MD (Medi-Lite score) and FRAX-derived fracture risk. A two-tailed *p*-value < 0.05 was considered statistically significant.

## 3. Results

**Figure 1:**
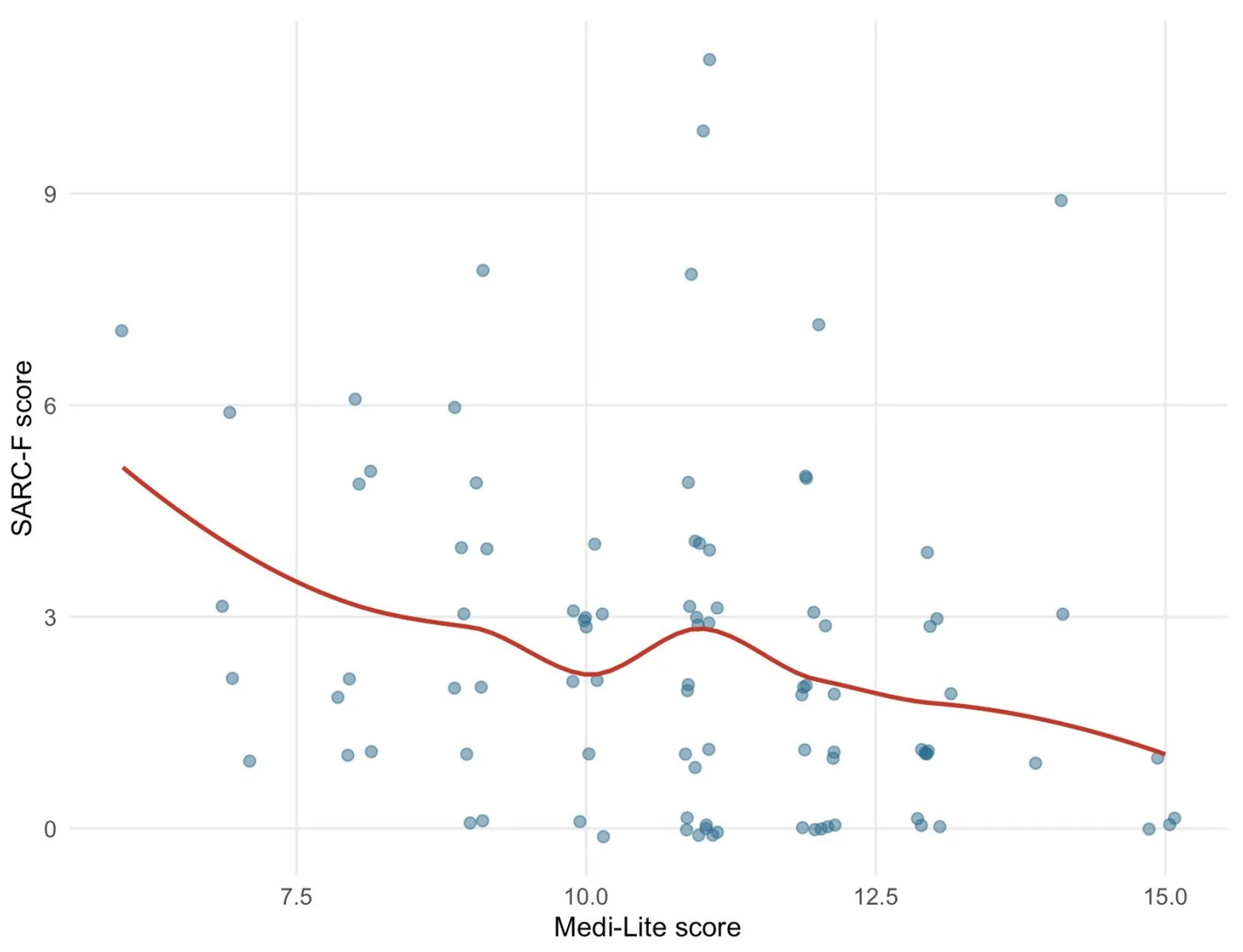
Association between Medi-Lite score and SARC-F score. Spearman correlation: r = −0.292, *p* = 0.005. Points are jittered to reduce overlap of discrete observations. The red line depicts a non-parametric LOESS-smoothed trend and is provided for visualization purposes only.

**Figure 2:**
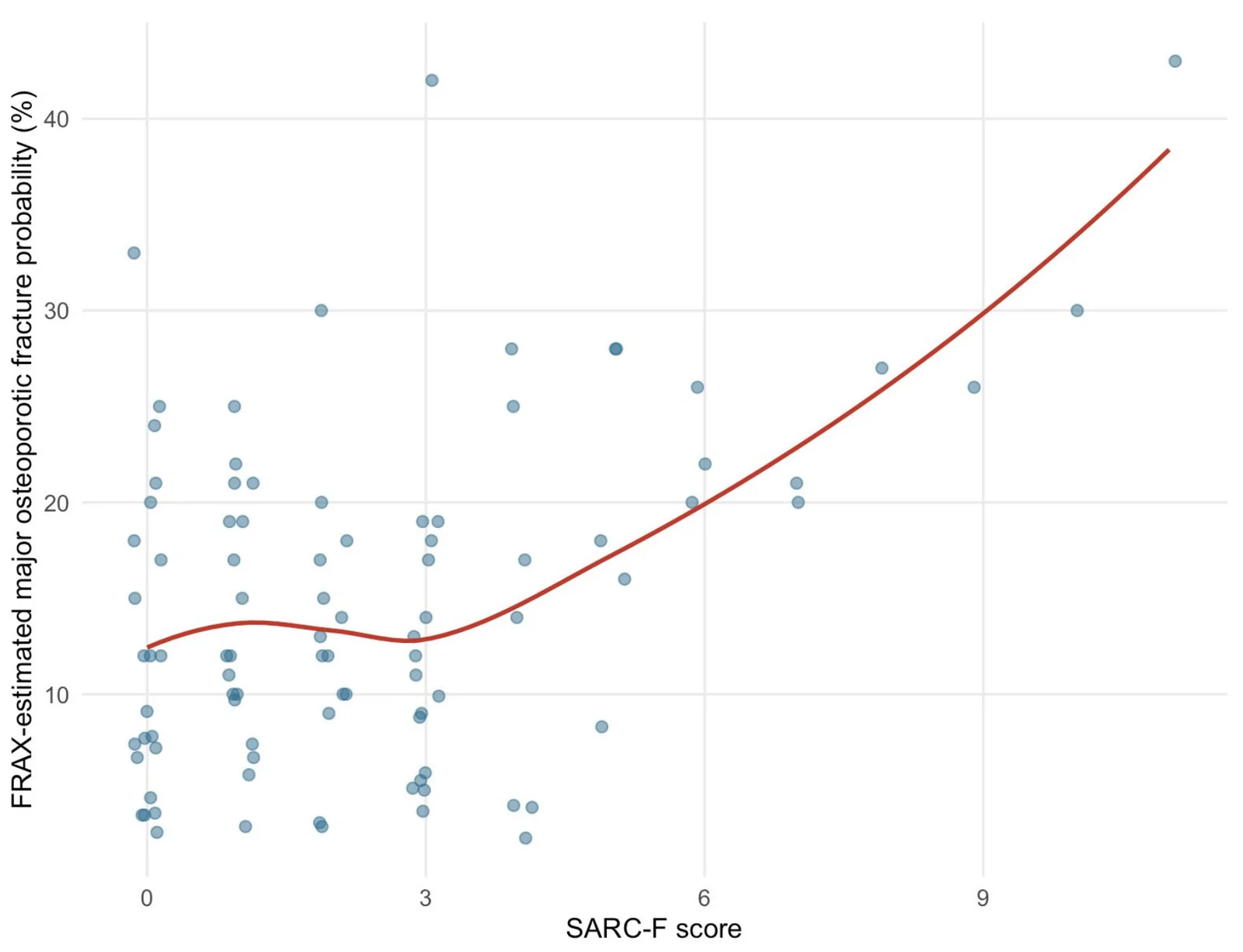
Association between SARC-F score and 10-year probability of major osteoporotic fracture assessed by FRAX. Spearman correlation: r = 0.289; *p* = 0.005. Points are jittered horizontally to reduce overplotting of discrete integer SARC-F scores. The red line represents a non-parametric (LOESS) smoothed trend, shown for descriptive purposes only.

A total of 96 postmenopausal women were included in the study. The median age was 66 years (interquartile range [IQR] 60.5–73), and the median body mass index (BMI) was 24.3 kg/m^2^ (IQR 21.9–26.9). The median 10-year probability of major osteoporotic fracture was 13% (IQR 7.9–20), while the median 10-year probability of hip fracture was 4% (IQR 1.4–9.9), as estimated by the FRAX algorithm. BMD values were consistent with osteopenia or osteoporosis; the median T-scores were −2.5 SD (IQR −3.1 to −1.8) at the lumbar spine and −2.2 SD (IQR −2.6 to −1.7) at the femoral neck. The median adherence to the MD, assessed by the Medi-Lite score, was 11 (IQR 10–12). The median SARC-F score was 2 (IQR 1–3), and the median handgrip strength was 20 kg (IQR 17.5–26) (Table 1). Age was positively correlated with SARC-F score (r = 0.370; *p* < 0.001), indicating a greater likelihood of impaired self-reported muscle-related functional status with increasing age. Adherence to the MD was inversely correlated with SARC-F score (r = −0.292; *p* = 0.005), suggesting better muscle-related functional status among participants with higher Medi-Lite scores (Figure 1). SARC-F score was inversely associated with handgrip strength (r = −0.264; *p* = 0.013), consistent with the expected relationship between higher SARC-F scores and lower objectively measured muscle strength; this modest association supports the plausibility of SARC-F as a surrogate marker of muscle performance but does not by itself establish its validity. Medi-Lite score was not significantly associated with handgrip strength, either unadjusted (r = −0.063; *p* = 0.55) or after adjustment for age and BMI (β = −0.03; *p* = 0.94). Twenty-three participants (24.5%) had a SARC-F score ≥ 4, and 19 participants (20.9%) had handgrip strength below 16 kg, which represents the EWGSOP2 consensus cut-off value for probable sarcopenia in women. Furthermore, SARC-F score was positively associated with the 10-year probability of major osteoporotic fractures (r = 0.289; *p* = 0.005) (Figure 2) and hip fractures (r = 0.277; *p* = 0.008), as estimated by the FRAX algorithm, indicating that poorer self-reported muscle-related functional status was associated with higher estimated fracture risk. To address the concern regarding potential confounding factors, we performed additional multivariate analyses adjusting for age and BMI. Notably, the associations of MD with SARC-F and of SARC-F with FRAX remained significant in multivariable regression analyses adjusted for age and BMI (Supplementary Table S1). Because age and BMI are themselves components of the FRAX algorithm, we additionally report the incremental R^2^ attributable to SARC-F: age and BMI alone explained R^2^ = 0.526 of the variance in FRAX-estimated major fracture probability, while adding SARC-F increased this to R^2^ = 0.565 (ΔR^2^ = 0.038; β = 0.79, 95% CI 0.23 to 1.35; *p* = 0.006). In a sensitivity analysis further adjusting for smoking status, previous fragility fracture, and family history of hip fracture, the association between Medi-Lite score and SARC-F remained significant (β = −0.35, *p* = 0.002); previous fragility fracture was independently associated with higher SARC-F score (β = 1.32, *p* = 0.003), while smoking and family history were not significant independent predictors. The association between the Medi-Lite score and SARC-F was materially unchanged when using a modified score excluding the alcohol component (r = −0.309, *p* = 0.0045 versus r = −0.291, *p* = 0.0044 for the original score).

When participants were stratified into tertiles of Medi-Lite adherence, age, BMI, BMD at both skeletal sites, handgrip strength, and FRAX-estimated probabilities did not differ significantly across groups (Kruskal–Wallis, all *p* > 0.15), whereas SARC-F score decreased significantly with increasing Medi-Lite tertile (*p* = 0.044), consistent with the correlation analysis. On the other hand, no significant associations were observed between adherence to the MD and FRAX-derived fracture risk, either for major osteoporotic fractures (r = 0.158; *p* = 0.129) or hip fractures (r = 0.141; *p* = 0.176). Similarly, adherence to the Mediterranean Diet was not significantly associated with BMD at either the lumbar spine or femoral neck, nor with fracture status. Similarly, adherence to the Mediterranean Diet was not significantly associated with BMD at the lumbar spine or femoral neck, nor with the occurrence of fragility fractures. Of the 43 participants with a history of fragility fracture, 21 (48.8%) presented at least one vertebral fracture according to the Genant semiquantitative classification, whereas 28 (65.1%) sustained a non-vertebral fragility fracture. Concomitant vertebral and non-vertebral fractures were observed in 6 participants, corresponding to 28.6% of those with vertebral fractures. The most common sites of non-vertebral fragility fractures were the wrist (*n* = 13) and ribs (*n* = 7), followed by the humerus (*n* = 2), sacrum (*n* = 2), pelvis (*n* = 2), and tibia (*n* = 1). Neither Medi-Lite score nor SARC-F score was significantly correlated with lumbar spine or femoral neck BMD or T-score (Medi-Lite: all |r| ≤ 0.14, *p* ≥ 0.21; SARC-F: all |r| ≤ 0.17, *p* ≥ 0.13). In an exploratory statistical decomposition (indirect-effect analysis) (5000 bootstrap samples, *n* = 96), the indirect effect of MD adherence on FRAX-derived major osteoporotic and hip fracture risk through SARC-F was significant (ACME = −0.33; 95% CI −0.75 to −0.05; *p* = 0.012; ACME = −0.28, 95% CI −0.66 to −0.03, *p* = 0.022, respectively), whereas the direct effect was not statistically significant (ADE = 0.58; 95% CI −0.02 to 1.22; *p* = 0.056; ADE = 0.10; 95% CI −0.52 to 0.65; *p* = 0.689, respectively). Because MD adherence, SARC-F, and FRAX-derived fracture risk were all assessed at the same time point, these findings are compatible with a possible indirect pathway involving SARC-F-assessed functional status, rather than demonstrating that diet adherence acts through, or is causally mediated by, muscle-related functional status; nonetheless temporal ordering and causality cannot be established from this cross-sectional design.

## 4. Discussion

In this monocentric cross-sectional study of Caucasian women in the postmenopausal period undergoing evaluation for osteoporosis, greater adherence to the MD was significantly associated with a more favorable muscle-related functional status, as reflected by lower SARC-F scores. In turn, poorer SARC-F-assessed functional status was significantly associated with higher estimated fracture risk, as assessed by the FRAX algorithm [34,35].

Collectively, these findings are consistent with the hypothesis that dietary patterns may influence fracture risk indirectly through functional pathways, possibly involving muscle status, rather than through direct effects on bone structure. Nevertheless, because dietary adherence, functional status, and fracture risk were assessed contemporaneously, no temporal or causal relationships can be inferred, and these observations should be regarded as hypothesis-generating.

The role of muscle function as a mediator of skeletal health has gained increasing recognition in recent years [5,7]. Reduced muscle strength and impaired physical performance are strongly associated with an increased risk of falls, which represent a major contributor to fragility fractures in older adults [4,5,6]. In this context, although SARC-F is primarily a screening tool and not a diagnostic instrument, it captures clinically meaningful aspects of functional decline and has been shown to predict adverse outcomes in aging populations, including disability, hospitalization, and mortality [44,45,46]. The modest inverse association observed in our cohort between SARC-F score and handgrip strength is consistent with, but does not by itself establish, SARC-F as a surrogate marker of muscle performance in this cohort. The association between higher adherence to the MD and better SARC-F-assessed functional status is biologically plausible and in accordance with an expanding body of literature supporting the role of healthy dietary patterns in preserving physical function [25,26].

The MD is characterized by a high intake of plant-based foods, extra virgin olive oil, and a balanced consumption of protein sources, resulting in a nutrient profile rich in antioxidants, anti-inflammatory compounds, and essential micronutrients [13,14,15,47]. These components may act synergistically to counteract age-related declines in muscle mass and function, potentially through attenuation of oxidative stress and chronic low-grade inflammation, which are recognized hallmarks of both sarcopenia and frailty [47,48,49].

Importantly, no significant associations were observed between MD adherence and BMD, prevalent fractures, or FRAX-derived fracture risk estimates. Likewise, SARC-F score was not associated with BMD or T-score at either skeletal site. Taken together, these findings suggest that the observed relationship between MD adherence, functional status, and FRAX-estimated fracture probability may be driven primarily by clinical risk factors incorporated into the FRAX algorithm rather than by an effect on bone density itself. However, this interpretation should be made with caution, as FRAX was calculated without BMD in the present study. It remains possible that the use of a BMD-inclusive FRAX estimate could yield different results, an issue that warrants investigation in future studies specifically designed to assess fracture risk using both clinical and densitometric parameters.

These results suggest that, within this specific clinical context, the influence of dietary patterns on musculoskeletal health may be mediated more strongly through functional rather than structural pathways.

This interpretation is further consistent with the exploratory statistical decomposition (indirect-effect analysis), which suggested that the association between adherence to the MD and FRAX-derived fracture risk is compatible with an indirect pathway involving muscle-related functional status, rather than a direct effect on skeletal parameters; however, because this analysis was based on cross-sectional data collected at a single time point, it should be interpreted as hypothesis-generating and cannot establish causality or temporal precedence. The observed positive associations between SARC-F scores and both major osteoporotic and hip fracture probabilities further suggest that muscle-related functional impairment may represent a relevant correlate of fracture susceptibility, independent of BMD [5,6,44,45,46].

Because age and BMI are themselves clinical inputs to the FRAX algorithm, the high R^2^ observed when regressing FRAX-estimated probability on these variables is partly expected by construction. SARC-F was associated with a modest but statistically significant increase in explained variance beyond age and BMI (ΔR^2^ = 0.038), suggesting a limited independent contribution of muscle-related functional status to FRAX-estimated probability, over and above its own embedded risk factors.

Overall, these findings are consistent with a growing body of evidence supporting a protective role of healthy dietary patterns, including the MD, in preserving musculoskeletal health in postmenopausal women [28,48], possibly through the attenuation of oxidative stress and chronic low-grade inflammation [24,25,26,27]. Emerging evidence further suggests that diet may influence musculoskeletal health through interactions with the gut microbiota. Indeed, microbiota composition and its metabolites, particularly short-chain fatty acids, have been implicated in the regulation of both muscle and bone metabolism, while greater adherence to the MD has been associated with a more favorable microbial profile [50,51]. In addition, dietary and phytotherapeutic interventions have been shown to modify microbial composition and metabolic activity, with consequent effects on intestinal barrier function and systemic inflammatory status [52,53]. Collectively, these observations provide further biological plausibility for a role of diet-mediated gut microbiota modulation in the maintenance of musculoskeletal and overall systemic health.

Although the gut-muscle-bone axis provides a biologically plausible explanation for the observed associations, this mechanism remains speculative in the context of the present study, as no direct assessment of gut microbiota composition or microbial metabolites was performed.

Several limitations of this study should be acknowledged. First, participants were recruited from a tertiary referral center specializing in bone and mineral metabolism disorders; consequently, the cohort may not be representative of the broader postmenopausal population and is likely characterized by a higher burden of skeletal fragility and functional impairment. Second, the cross-sectional design precludes causal inference, and longitudinal studies are needed to clarify the temporal relationships among dietary patterns, muscle function, and fracture risk. Third, although SARC-F is a practical and widely used screening tool, it does not directly assess muscle mass and cannot establish a diagnosis of sarcopenia according to current consensus criteria. Fourth, fracture risk was assessed using FRAX-derived 10-year probability estimates rather than prospectively recorded fracture events, thereby limiting conclusions regarding actual fracture incidence. Finally, although statistically significant, the observed associations were modest in magnitude (e.g., the correlation between Medi-Lite and SARC-F scores explained approximately 8.5% of the shared variance) and should therefore be interpreted as exploratory and hypothesis-generating rather than as evidence of strong clinical effects.

Furthermore, residual confounding cannot be entirely excluded. Several potentially relevant variables, including socioeconomic status, educational level, depression, cognitive status, pain, osteoarthritis, comprehensive medication use, vitamin D and calcium supplementation, anti-osteoporosis treatment, and time since menopause, were not systematically collected and therefore could not be included in the analyses. Physical activity was assessed only qualitatively through clinical interview and showed limited variability across participants, precluding its inclusion as a covariate in the multivariable models. Although participants were not sedentary, none reported regular engagement in structured or vigorous exercise programs. Because physical activity may independently influence both dietary adherence and functional status, some degree of residual confounding by this factor cannot be excluded. Future studies should incorporate validated measures of physical activity to allow formal adjustment.

In addition, the sample size calculation was based on the detection of a moderate correlation and was not specifically designed to support mediation analyses, which generally require larger samples to achieve stable bootstrap-derived confidence intervals. Consequently, the mediation findings should be considered preliminary and hypothesis-generating. Moreover, because the indirect-effect analysis was conducted using cross-sectional data, neither causal mediation nor temporal precedence can be established, and the results should be interpreted with appropriate caution.

Despite these limitations, the study has several strengths, including the use of validated instruments to assess dietary adherence, muscle function, and fracture risk, as well as the focus on a clinically relevant population at high risk of skeletal fragility. The integration of nutritional and functional parameters provides a comprehensive perspective on musculoskeletal health, with potential implications for both clinical practice and future research.

## 5. Conclusions

In conclusion, greater adherence to the MD was associated with a more favorable functional status, as assessed by the SARC-F questionnaire, in this cross-sectional cohort of postmenopausal women referred for osteoporosis evaluation. Moreover, poorer functional status was associated with higher FRAX-derived fracture risk estimates. Collectively, these findings are consistent with the hypothesis that muscle-related functional status may represent an intermediate link between dietary habits and estimated skeletal fragility. However, because dietary adherence, functional status, and fracture risk were assessed simultaneously, the cross-sectional design precludes any inference regarding causality or temporal sequence. Accordingly, the observed pathway should be regarded as preliminary and hypothesis-generating, requiring confirmation in adequately powered longitudinal studies. From a clinical perspective, these findings support the potential relevance of dietary strategies aimed at preserving musculoskeletal function in postmenopausal women at increased risk of osteoporosis and fracture. Future prospective and mechanistic studies are warranted to further elucidate the complex interplay among nutrition, muscle function, and skeletal health.

## Figures and Tables

**Table 1:** Main clinical characteristics of participants.

Variable	Postmenopausal Women (*n* = 96)
Age (yr.)	66 (60.5 to 73)
BMI (kg/m^2^)	24.3 (21.9 to 26.9)
10-yr probability of major fracture (%)	13 (7.9 to 20)
10-yr probability of hip fracture (%)	4 (1.4 to 9.9)
Lumbar spine BMD (g/cm^2^)	0.8 (0.7 to 0.85)
Lumbar spine T-score (SD)	−2.5 (−3.1 to −1.8)
Femoral neck BMD (g/cm^2^)	0.62 (0.56 to 0.67)
Femoral neck T-score (SD)	−2.2 (−2.6 to −1.7)
Osteoporosis *n* (%)	57 (59.4)
Osteopenia *n* (%)	38 (39.6)
SARC-F score	2 (1 to 3)
SARC-F ≥ 4 *n* (%)	23 (24)
Handgrip strength (kg)	20 (17.5 to 26)
Handgrip < 16 kg *n* (%)	19 (19.8)
Medi-Lite score	11 (10 to 12)
Family history of hip fracture *n* (%)	56 (58.3)
Previous fragility fracture *n* (%)	43 (44.8)
Smoking *n* (%)	25 (26.0)
Alcohol intake ≥ 3 units/day *n* (%)	0 (0)
Glucocorticoid use *n* (%)	0 (0)
Rheumatoid arthritis *n* (%)	1 (1)
Secondary osteoporosis *n* (%)	4 (4.2)

Continuous variables are presented as median (interquartile range); categorical variables are presented as *n* (%).

## Data Availability

The original contributions presented in this study are included in the article. Further inquiries can be directed to the corresponding authors.

## Supplementary Materials

The following supporting information can be downloaded at: https://www.mdpi.com/article/10.3390/nu18162629/s1, Supplementary Table S1. Multiple linear regression analyses examining the associations of adherence to the Medi-terranean diet, age, body mass index (BMI), and SARC-F score with sarcopenia risk (SARC-F) and fracture risk assessed by FRAX (for major osteoporotic fracture and hip fracture) in the study population (*n* = 96).
