# Supplementary material for: Association of Mediterranean Diet Adherence with Self-Reported Muscle-Related Functional Status and Fracture Probability in Postmenopausal Women Evaluated for Osteoporosis: A Cross-Sectional Study
Source: Nutrients. 2026 Aug 12;18(16):2629. doi: 10.3390/nu18162629 (PMC13516989; doi:10.3390/nu18162629)
Supplement: Supplementary file 1 [file nutrients-18-02629-s001.zip › nutrients-4439270-supplementary.pdf]

Supplementary Table S1.

S1.1. Outcome: SARC-F (n = 96; R^2 = 0.25)

Predictor	β (standardized)	B (95% CI)	p-value
MEDI-LITE	-0.30	-0.352 (-0.569 to -0.134)	0.002
AGE	0.43	0.114 (0.064 to 0.163)	<0.001
BMI	0.10	0.052 (-0.045 to 0.149)	0.288

S1.2. Outcome: FRAX major fracture (n = 96; R^2 = 0.58)

Predictor	β (standardized)	B (95% CI)	p-value
MEDI-LITE	0.13	0.580 (-0.071 to 1.231)	0.080
SARC-F	0.25	0.939 (0.340 to 1.538)	0.002
AGE	0.60	0.594 (0.439 to 0.749)	<0.001
BMI	-0.17	-0.342 (-0.617 to -0.067)	0.015

S1.3. Outcome: FRAX hip fracture (n = 96; R^2 = 0.50)

Predictor	β (standardized)	B (95% CI)	p-value
MEDI-LITE	0.02	0.095 (-0.546 to 0.736)	0.769
SARC-F	0.23	0.794 (0.204 to 1.384)	0.009
AGE	0.53	0.476 (0.323 to 0.628)	<0.001
BMI	-0.29	-0.509 (-0.779 to -0.238)	<0.001

Baseline model (age + BMI only): R^2 = 0.526
Incremental R^2 from adding SARC-F to the age + BMI baseline model: ΔR^2 = 0.038 (β = 0.79, 95% CI 0.23–1.35, p = 0.006)

β = standardized regression coefficient. B = unstandardized regression coefficient. Confidence intervals refer to the unstandardized regression coefficients.
